# Purification of Cyclic GMP-AMP from Viruses and Measurement of Its Activity in Cell Culture

**DOI:** 10.1007/978-1-4939-7237-1_8

**Published:** 2017-08-15

**Authors:** Alice Mayer, Jonathan Maelfait, Anne Bridgeman, Jan Rehwinkel

**Affiliations:** 3 Medical Research Council Human Immunology Unit, Radcliffe Department of Medicine, Medical Research Council Weatherall Institute of Molecular Medicine, University of Oxford, John Radcliffe Hospital, Headley Way, Oxford, OX3 9DS UK; 4 Medical Research Council Human Immunology Unit, Radcliffe Department of Medicine, Medical Research Council Weatherall Institute of Molecular Medicine, University of Oxford, Headley Way, Oxford, OX3 9DS UK

**Keywords:** Innate immunity, cGAMP, STING, Type I IFN, Bioassay

## Abstract

Sensing of cytoplasmic DNA by cGAS is essential for the initiation of immune responses against several viruses. cGAS also plays important roles in some autoinflammatory and autoimmune diseases and may be involved in immune responses targeting cancer cells. Once activated, cGAS catalyzes the formation of the di-nucleotide 2′-3′-cyclic GMP-AMP (cGAMP), which propagates a signaling cascade leading to the production of type I interferons (IFNs). Interestingly, cGAMP is incorporated into enveloped viruses and is transferred to newly infected cells by virions. In this article, we describe a method to purify cGAMP from viral particles and a bioassay to measure its activity. This assay takes advantage of a reporter cell line that expresses the genes encoding green fluorescent protein (GFP) and firefly luciferase under the control of the IFNß promoter, allowing the testing of several samples in a single experiment taking not more than 3 days.

## Introduction

Sensing of foreign DNA playsVirus infection a central role in the detection of viral infections by the immune system. In healthy cells, DNA is restricted to specialized compartments, namely the nucleus and mitochondria. Presence of DNA in other subcellular compartments is detected by specific receptors and triggers different signaling cascades, some of which culminate in the secretion of type I IFNs. These cytokines in turn act on the infected cell and on neighboring cells to induce an antiviral state, leading to a reduction in viral replication and spread. Type I IFNs also play roles in the activation of the adaptive immune response and therefore are crucial to the successful defence of the host against viruses.

In 2013, Sun et al. discovered that the presence of DNA in the cytosol is sensed by the cyclic GMP-AMP synthase (cGAS) [1]. Instead of signaling via protein-protein interactions as often seen for other sensors of virus presence, cGAS—once activated—catalyzes the synthesis of the di-nucleotide 2′-3′-cyclic GMP-AMP (hereafter simply Bioassay). cGAMP acts as a second messenger and activates the endoplasmic reticulumEndoplasmic reticulum (ER)-bound secondary receptor STING [2]. Interestingly, STING is also activated by the bacterial dinucleotides cyclic-di-GMP and cyclic-di-AMP [3, 4]. The interaction of cyclic di-nucleotides with STING induces a change of its conformation, which results in recruitment and activation of the kinase TBK1 and the transcription factor IRF3. Once phosphorylated by TBK1, IRF3 dimerizes and translocates to the nucleus to induce the expression of type I IFNs. An interesting feature of this signaling pathway is that cGAMP—being a small molecule—can diffuse from cell to cell via gap junctions [5]. This allows rapid propagation of the signal in cells connected by gap junctions as soon as one cell is infected. Another consequence of cGAMP’s nature as a small, diffusible molecule is that it can be incorporated into enveloped viral particles during budding and is transferred from one cell to another cell by viruses [6, 7]. This may allow newly infected cells to respond faster during secondary rounds of infection.

The cGAS pathway not only plays a critical role in the initiation of immune responses against several DNA viruses (including Herpes viruses, Vaccinia virus, adenovirus, Hepatitis B virus, mouse, and human cytomegaloviruses), retroviruses (including HIV), and bacteria (including *Mycobacterium tuberculosis*), but also in antitumor immunity [8, 9]. Of note, injection of cGAMP directly into solid tumors enhances the immune response targeting both the injected tumor and distal tumors [10, 11]. Moreover, cGAMP has been successfully used as an adjuvant in several vaccination models [12–14]. However, uncontrolled activation of cGAS by endogenous ligands can also lead to the development of inflammatory pathologies such as Aicardi-Goutières Syndrome [8, 9].

Because of the central role of cGAS in the development of immune responses in a broad range of pathological conditions, measuring its product, cGAMP, is important in studies aimed at understanding the physiopathology of infectious and inflammatory diseases. The methods currently available to detect cGAMP are (1) reverse phase HPLCHigh pressure liquid chromatography (HPLC) followed by tandem mass spectrometry analysis [2, 6, 7, 15, 16] or by NMR spectroscopy [15, 17, 18] and (2) bioassays in which the activation of STING is assessed after incubation of mildly permeabilized cells with samples containing cyclic dinucleotides [2, 3]. The first approach may be more sensitive, but it also requires specialized equipment and expertise and has limitations in terms of the number of samples that can be processed. Bioassays have the advantage of being fast and requiring only standard tissue culture facilities, and can also be adapted to large sample numbers. Importantly, bioassays also provide information on the biological activity of the tested samples, and can therefore be used not only to detect the presence of cGAMP, but also to test the ability of different cellular, viral, or synthetic components to modulate STING signaling.

**Fig. 1:**
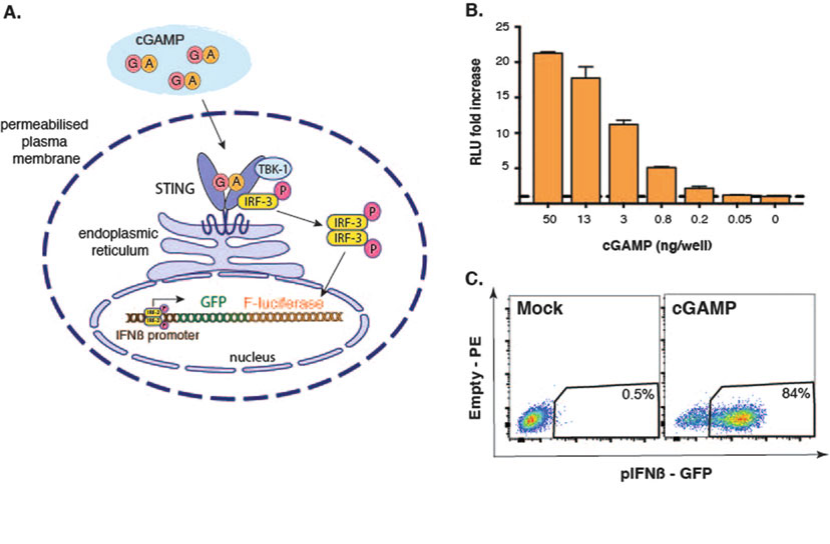
(**a**) IllustrationVirus infection of the bioassay. (**b**) Representative luciferase assay: PMA-treated p125-THP1 cells were stimulated with graded doses of cGAMP. Luciferase activity was measured 24 h post simulation. Data represent the fold increase of firefly luciferase activity relative to the unstimulated mock control. Error bars indicate SD from four technical replicates. (**c**) Representative FACS plots: GFP expression in PMA-treated cells stimulated for 6 h with 25 ng of cGAMP per well or mock treated (gated on DAPI-negative single cells)

In this chapter, we describe a step-by-step protocol to purify Bioassay from viral particles and a bioassay to detect and quantify its activity. The method for small molecule extraction from virions was adapted from [16]. Briefly, viral particles are lysed in a buffer containing 1% Triton X-100 and nucleic acids are degraded by the treatment with an endonuclease that degrades both DNA and RNA (benzonase). Proteins are then removed by two successive rounds of phenol-chloroform extraction, followed by a chloroform wash to remove all traces of phenol. The extract is then filtered through a 3 kDa centrifugal filter and the filtrate is concentrated by centrifugation under vacuum. With this method, we typically recover 36% of the cGAMP present in the original sample. The bioassay used to quantify cGAMP activity has been adapted from [2, 3] and takes advantage of a reporter cell line we have generated. These cells were derived from the monocytic cell line THP-1 and express firefly luciferase and GFP under the control of the human IFNß promoter. Overnight incubation in the presence of PMA induces the differentiation of these THP-1 reporter cells into macrophage-like cells. This step is important to increase the sensitivity to stimulation with cGAMP. The bioassay involves incubation of the cells with samples diluted in an isotonic buffer containing a very low concentration of digitonin. This mild detergent causes the formation of small holes in the plasma membrane, such that small molecules can diffuse into the cell but proteins and intracellular organelles stay in place (Fig. 1a). After half an hour, the stimulus is washed away and cells are incubated in normal media for 6–24 h, at which point firefly luciferase activity or GFP fluorescence is measured with a luminometer (Fig. 1b) or by flow cytometry (Fig. 1c), respectively. The lower detection limit of this bioassay using luciferase as readout typically ranges from 0.05 to 0.2 ng per well (2–8 ng/mL, Fig. 1b). Taken together, this bioassay allows the measurement of cGAMP concentration in several samples in only 3 days.

## Materials

### cGAMP

1. X-100 lysis buffer: 1 mM NaCl, 3 mM MgCl2, 1 mM EDTA, 1% Triton X-100, 10 mM Tris ph 7.4.
2. Optional: (2′-3′) cGAMP: Cyclic (guanosine- (2′ − > 5′)- monophosphate- adenosine- (3′ − > 5′)-monophosphate (Biolog or Invivogen; *see* **Note** **1**).
3. Benzonase.
4. P:I:C [phenol:chloroform:isoamyl alcohol 25:24:1].
5. Chloroform.
6. Amicon Ultra 3 K filter.
7. Sterile nuclease-free distilled water.
8. Ultracentrifuge to pellet viruses and associated tubes.
9. Standard table top centrifuge.
10. Chemical hood for handling of phenol-chloroform.
11. Speed vac to dry samplescGAMP.

### Bioassay Bioassay

#### Seeding and Differentiation of THP-1 cGAMPReporter Cells

1. Cells: p125-THP1 clone 9. These reporter cells were generated as follows: THP-1 cells were lentivirally transduced with a construct coding for GFP and firefly luciferase under control of the human IFNß promoter and were then cloned by limiting dilution (cells available upon request, *see* **Note** **2**).
2. R10 media: 1× RPMI media, 10% fetal calf serum (FCS), 2 mM l-glutamine, 50 μM 2-mercapto-ethanol.
3. PMA (Phorbol 12-myristate 13-acetate).
4. Sterile flat-bottom 96-well plates.
5. Basic tissue-culture devices: centrifuge, 5% CO_2_ incubator, laminar flow hood, material for counting cells, 50 mL conical tubes, pipettes and multichannel pipettescGAMP.

#### Stimulation of THP-1 Reporter Cells

1. R10 media and basic tissue-culture devices (hood, centrifuge, incubator, etc.).
2. Optional: sterile v-bottom 96-well plates.
3. 2× permeabilization (2xPERM) buffer: 100 mM Hepes-HCl (pH 7.4), 200 mM KCl, 6 mM MgCl2, 0.4% BSA, 170 mM sucrose, 2 mM ATP, 0.2 mM GTP, 0.002% digitonin (*see* **Note 3**).
4. Sterile nuclease-free distilled water.
5. (2′-3′) cGAMP: Cyclic (guanosine-(2′ − > 5′)-monophosphate- adenosine-(3′ − > 5′)-monophosphate (*see* **Note** **1**).

#### Luciferase Assay

1. R10 media and multichannel pipettes.
2. OneGlo luciferase assay system.
3. Optiplate 96-white microplate.
4. LuminometerLuminometer Luciferase Assay.

## Methods

### cGAMP

1. Resuspend pelleted viruses in 500 μL of X-100 lysis buffer, transfer to 1.5 mL tubes, and incubate 20 min on ice, vortex regularly (*see* **Note** **4**).
2. Centrifuge for 10 min at 1000 × *g* at 4 °C.
3. Optional: Spike 1 μg cGAMP into 500 μL X-100 lysis buffer as a positive control (to test the efficiency of the purification process).
4. Collect the supernatant, add 50 U/mL of benzonase and incubate for 45 min on ice.
5. Add 500 μL of P:I:C, vortex vigorously, spin for 5 min at 17,000 × *g* (*see* **Note** **5**).
6. Take off upper aqueous layer by pipetting carefully without disturbing the lower layer.
7. Add 500 μL P:I:C, vortex vigorously, spin 5 min at 17,000 × *g*.
8. Take off upper aqueous layer by pipetting carefully without disturbing the lower layer.
9. Add 500 μL chloroform, vortex vigorously, spin 5 min at 17,000 × *g*.
10. Transfer the upper aqueous layer onto Amicon 3 K filter column and centrifuge 30′ at 14,000 × *g*.
11. Dry the samples using a Speed Vac, resuspend pellets in 20 μL H_2_O, and store at −80 °CcGAMP.

### Bioassay

#### Seeding and Differentiation of THP-1 Reporter Cells (Day 0 of the Bioassay

50,000 p125-THP1 cells are seeded per well in the presence of 5 ng/mL of PMA. *See* **Note** **6** for an estimation of the number of wells to seed.

1. Harvest the THP-1 reporter cells, centrifuge, resuspend in R10 media, and count.
2. Adjust the cell concentration to 5 × 10^5^ cells per mL.
3. Add PMA to a final concentration of 5 ng/mL.
4. Dispatch 100 μL per well in a flat-bottom 96-well plate.
5. Place in a tissue culture incubator (37 °C, 5% CO_2_) and incubate overnight (*see* **Note** **7**).

#### Stimulation of THP-1 Reporter Cells (Day 1 of the Bioassay

1. Warm some R10 media to 37 °C and bring nuclease-free distilled water to room temperature.
2. Thaw the 2× PERM buffer, the samples containing the cGAMP to dose and some cGAMP for the standard.
3. Dilute the cGAMP-containing samples in nuclease-free distilled water to a total of 55 μL for duplicate measurements or 80 μL for triplicates (see **Notes** **8** and **9**).
4. Further dilute samples 1:2 with 2× PERM buffer, then titer down in twofold dilution series in 1× PERM buffer. We usually do between 4 and 6 dilutions per sample (*see* **Note** **6**).
5. Dilute the (2′-3′) cGAMP standard in 1× PERM buffer, from 50 ng/well to 0.02 ng/well in two-fold dilution series. Prepare 80 μL of each dilution (triplicates). Do not forget to keep 80 μL of 1× PERM buffer only as a negative control (blank).
6. Wash the reporter cells by removing the medium and by replacing it with 100 μL of fresh R10 (*see* **Note** **10**).
7. Remove all medium.
8. Gently overlay the cells with 25 μL of sample or standard dilutions.
9. Incubate for 30 min in a tissue culture incubator.
10. Wash the cells by adding 100 μL of fresh R10 per well, and then remove all medium.
11. Add 100 μL of fresh R10 media and incubate between 6 and 24 h in a tissue culture incubatorBioassay.

#### Luciferase Assay (Day 2 of the Bioassay, See Note 11)

1. Dilute the One Glo reagent 1:2 with R10 media, protect from light, and wait until it reaches room temperature.
2. Flick off the media from the plates containing the THP1 cells and replace with 100 μL of the diluted One Glo reagent.
3. Incubate for 3 min at room temperature in the dark.
4. Pipette up and down to homogenize and transfer 75 μL to a white 96-well plate.
5. Read with a luminometer according to the manufacturer’s instructionsLuciferase Assay Bioassay.

## Notes

1. It is important to use cGAMP molecules with a (2′-5′) link between the guanosine and the adenine and a (3′-5′) link between the adenine and the guanosine (here referred to as (2′-3′) cGAMP). cGAMP with (3′-5′) links in both positions has a lower affinity for human STING and is not as efficient at inducing its activation [15–18].
2. The growth of p125-THP1 clone 9 is similar to parental, unmodified THP-1 cells. These cells are suspension cells grown in R10. We suggest passaging them by diluting cells once or twice per week by adding fresh R10 media. Typically, we dilute these cells 1:3 to 1:5 when they reach 10^6^/mL. We do not split them if there are less than 6 × 10^5^ cells/mL. This clone is available upon request.
3. The 2× PERM buffer can be made in advance, filtered, aliquoted, and stored at −20 °C.
4. We typically analyze pelleted virus stocks corresponding to at least 10^6^ infectious units, although this will depend on the type of virus and the amount of cGAMP incorporated during budding. For highly concentrated samples, it is advisable to increase the volume of X-100 lysis buffer, in which case the volumes of P:I:C and chloroform need to be adjusted accordingly. It is noteworthy that we have not been able to detect cGAMP activity in extracts when using this protocol to recover cGAMP from DNA-stimulated cells. Nevertheless, it may be possible to use this method to purify cGAMP from cells overexpressing cGAS [16].
5. All the steps involving P:I:C and chloroform should be performed in a chemical hood. These reagents, and also the tubes and pipette tips that have been in contact with them, should be disposed of in an appropriate way. Phase lock tubes can be used for these extractions.
6. The number of wells to seed depends on the number of samples, the number of dilutions of each sample, and the number of technical replicates (ideally triplicates). The number of dilutions depends on the expected concentration of cGAMP in the sample, taking into account that the assay usually saturates around 10 or 20 ng/well. We typically analyze between 4 and 6 dilutions per sample. Ideally, 30 wells for the standard (9 dilutions and a blank, all in triplicate) and 18 wells per sample are required.
7. The cells should be incubated with PMA for at least 18 h, and this incubation can be extended up to 24 h. Shorter and longer incubation periods have not been tested with the luciferase readout; however, using flow cytometry, 12 h or 42 h incubation gave rise to a diminished fraction of GFP-positive cells.
8. We typically prepare the dilutions of the standard and the samples in sterile v-bottom 96-well plates, and then transfer these to the cells with a multichannel pipette.
9. We typically use only half of our samples (10 μL). If the sample contains a concentration of cGAMP very close to the lower detection limit, we suggest using all of it without serial dilution. In this case, add 7.5 μL of water to the 20 μL of sample and do the experiment in duplicate.
10. In all the wash steps, media can be removed with a multichannel pipette or flicked off the plate.
11. The activation of the IFNß promoter can also be assessed by flow cytometry. This method takes more time, but is an alternative if you do not have access to the equipment needed for luciferase assays. In that case, the cells should be harvested 6 h after stimulation as follows: (1) flick off the supernatant and add 200 μL of ice-cold FACS buffer (PBS, 2 mM EDTA, 1% FCS, and 0.02% sodium azide), (2) incubate for a minimum of 2 minutes on ice, (3) detach the cells by pipetting up and down and transfer to a v-bottom 96-well plate, (4) centrifuge 5 min at 500 × *g*, (5) flick off the supernatant and resuspend the cells in 100 μL of FACS buffer containing 1 ng/mL of DAPI. The fold increase of the GFP median fluorescence intensity (gated on DAPI-negative cells) is comparable to the luciferase assayluciferase assay.
